# Supplementary material for: Can knowledge reclassification accelerate technological innovation?
Source: PLoS One. 2026 May 18;21(5):e0348804. doi: 10.1371/journal.pone.0348804 (PMC13183286; doi:10.1371/journal.pone.0348804)
Supplement: S1 Appendix — (PDF) [file pone.0348804.s001.pdf]

Supporting Information Appendix

Peter Persoon [1,2*¤]

1 Oxford Martin School, University of Oxford, United Kingdom
2 Wageningen University and Research, Wageningen, The Netherlands

¤Current Address: Wageningen University and Research, Droevendaalsesteeg 4, 6708 PB Wageningen
* peter.persoon@wur.nl

S1 Appendix: Analytical model solutions

In this first supporting information appendix, I include detailed derivations of the analytical model. I also include a discussion of the possibility of negative net reclassifications and how to calculate the number of classifications per family. First I quickly repeat the model terminology. For some technological class, let me denote the number of families with filing year c at time t by $n_c(t)$ (i.e. the 'cohorts') and $n(t) = \sum_c n_c(t)$. For convenience, let $t = 0, 1, 2, 3, ..$ and $c = 0, 1, 2, 3, ...$ The solution should satisfy

$$\Delta_t n_c(t) = \begin{cases} 0 & \text{if } c > t+1, \\ \alpha n(t) & \text{if } c = t+1, \\ \beta n_c(t)/(t-c+1) & \text{if } c < t+1, \end{cases} \tag{1}$$

with constants $\alpha \geq 0$ and $\beta \geq 0$. I focus on the simplest case, a technology with one family at $t = 0$ and $c = 0$, i.e. $n_c(0) = 1$ for $c = 0$ and $n_c(0) = 0$ otherwise, equivalently, in terms of the Kronecker δ-function, $n_c(0) = \delta_{0,c}$.

Exact solution for cohorts

Let me first derive an exact solution for the number of patents in each cohort over time, i.e. $n_c(t)$. By definition, the binomial coefficient satisfies $\binom{x+1}{y+1} - \binom{x}{y} = \binom{x}{y+1}$ and $\binom{x}{y} = \frac{x-y+1}{y}\binom{x}{y-1}$, so I can write, for $t > c$

$$\binom{t-c+\beta+1}{t-c+1} n_c(c) - \binom{t-c+\beta}{t-c} n_c(c) = \binom{t-c+\beta}{t-c+1} n_c(c) \tag{2}$$

$$= \frac{t-c+\beta-t+c-1+1}{t-c+1}\binom{t-c+\beta}{t-c} n_c(c) \tag{3}$$

$$= \frac{\beta}{t-c+1}\binom{t-c+\beta}{t-c} n_c(c). \tag{4}$$

Say $n_c(t) = \binom{t-c+\beta}{t-c} n_c(c)$, on the left-hand side of Equation 2 I then recognize $\Delta_t n_c(t) = n_c(t+1) - n_c(t)$ and on the right-hand side of Equation 4 I recognize $\beta n_c(t)/(t-c+1)$. Together, these correspond to the third line of Equation 1. Given that $\binom{x}{y} = 0$ for $y < 0$, the relation $n_c(0) = \delta_{0,c}$ is also satisfied, and likewise for the

first line of Equation 1. Note this implies that $n_c(t) = 0$ for all $t \leq c$. Finally, in order to satisfy the second line of Equation 1, I require that

$$\alpha n(c-1) = n_c(c) - n_c(c-1), \tag{5}$$
$$= n_c(c). \tag{6}$$

This in turn implies that

$$n_c(c) = \alpha \sum_{c'=0}^{c-1} n_{c'}(c-1), \tag{7}$$
$$= \alpha \sum_{c'=0}^{c-1} \binom{c-1-c'+\beta}{c-1-c'} n_{c'}(c'), \tag{8}$$

which is a recursive relation involving $n_c(c)$ only. To solve this relation I will use an identity and an ansatz. The identity I use comes from Concrete Mathematics (page 202) Equation 5.62 [1] and says that for each integer n, any real r,s,t, and summing over all k

$$\sum_k \binom{tk+r}{k}\binom{tn-tk+s}{n-k}\frac{r}{tk+r} = \binom{tn+s+r}{n}, \tag{9}$$

which for $t = 1$ reduces to

$$\sum_k \binom{k+r-1}{k}\binom{n-k+s}{n-k} = \binom{n+s+r}{n}. \tag{10}$$

Alternatively, if $\beta = r - 1$, $n = c - 1 - u$ and $s = u\beta - 1 + u$, I can write it as

$$\sum_k \binom{k+\beta}{k}\binom{c+u\beta-2-k}{c-1-u-k} = \binom{(u+1)\beta+c-1}{c-u-1}. \tag{11}$$

Plugging in Equation 8 the ansatz $n_c(c) = \sum_{u=0}^{c} \binom{u\beta+c-1}{c-u}\alpha^u$ and using the earlier mentioned identity in the second to last step, I obtain for $c > 0$

$$\sum_{u=0}^{c} \binom{u\beta+c-1}{c-u}\alpha^u = \alpha \sum_{c'=0}^{c-1} \binom{c-1-c'+\beta}{c-1-c'} \sum_{u=0}^{c'} \binom{u\beta+c'-1}{c'-u}\alpha^u, \tag{12}$$
$$= \alpha \sum_{c'=0}^{c-1}\sum_{u=0}^{c-1} \binom{c-1-c'+\beta}{c-1-c'}\binom{u\beta+c'-1}{c'-u}\alpha^u, \tag{13}$$
$$= \alpha \sum_{u=0}^{c-1}\sum_{c'=0}^{c-1} \binom{c-1-c'+\beta}{c-1-c'}\binom{u\beta+c'-1}{c'-u}\alpha^u, \tag{14}$$
$$= \alpha \sum_{u=0}^{c-1}\left(\sum_{k=0}^{c-1} \binom{k+\beta}{k}\binom{u\beta+c-2-k}{c-1-u-k}\right)\alpha^u, \tag{15}$$
$$= \alpha \sum_{u=0}^{c-1} \binom{(u+1)\beta+c-1}{c-1-u}\alpha^u, \tag{16}$$
$$= \sum_{u=1}^{c} \binom{u\beta+c-1}{c-u}\alpha^u, \tag{17}$$

The left- and right-hand side of Equation 17 only disagree in case $c = 0$, but since the ansatz satisfies $n_0(0) = \binom{-1}{0} = 1$, by plugging in the ansatz in $n_c(t) = \binom{t-c+\beta}{t-c}n_c(c)$, I

obtain the exact solution for all c

$$n_c(t) = \binom{t-c+\beta}{\beta} \sum_{u=0}^{c} \binom{u\beta + c - 1}{c-u} \alpha^u. \tag{18}$$

Since $n_t(t) = \alpha n(t-1)$, I have also derived an expression for $n(t)$ for $t \geq 1$,

$$n(t-1) = \frac{1}{\alpha} \sum_{u=0}^{t} \binom{u\beta + t - 1}{t-u} \alpha^u \tag{19}$$

$$\tag{20}$$

or, for $t \geq 0$

$$n(t) = \frac{1}{\alpha} \sum_{u=0}^{t+1} \binom{u\beta + t}{t-u+1} \alpha^u \tag{21}$$

$$= \sum_{u=0}^{t+1} \binom{(u-1+1)\beta + t}{t-(u-1)} \alpha^{u-1} \tag{22}$$

$$= \sum_{u=-1}^{t} \binom{(u+1)\beta + t}{t-u} \alpha^u \tag{23}$$

$$= \sum_{u=0}^{t} \binom{(u+1)\beta + t}{t-u} \alpha^u \tag{24}$$

Determine growth factor

In Figure 4 in the main text, I plot $n_c(t)$ for several t on the left and $n(t)$ for several values of α and β on the right. It is clear that for large t, the number of families increases exponentially, with a base tending to a constant g that depends both on α and β. Let me first look at two extremes: say $\beta = 0$, then summing Equation 1 left and right over all c gives $\Delta_t n(t) = \alpha n(t)$ which directly suggest $n(t) \propto (1+\alpha)^t$. Let me consider another extreme where in the third line of Equation 1 I instead have the expression $\beta n_c(t)$, which is always larger than the original expression $\beta n_c(t)/(t-c+1)$ and therefore gives a larger estimation of $n(t)$. Again summing left and right over all c would then give $\Delta_t n(t) = (\alpha + \beta) n(t)$ which directly suggests $n(t) \propto (1+\alpha+\beta)^t$. Therefore, I have that $1 + \alpha \leq g < 1 + \alpha + \beta$.

To obtain a more precise expression for g, it is instructive to calculate the generating function $G(z)$ of $n(t)$, that is, $G(z) = \sum_{t=0}^{\infty} n(t) z^t$ for some $0 \leq z < 1$. Using the

expression for $n(t)$ in Equation 24, I obtain

$$G(z) = \sum_{t=0}^{\infty} \sum_{u=0}^{t} \binom{(u+1)\beta + t}{t-u} \alpha^u z^t, \tag{25}$$

$$= \sum_{u=0}^{\infty} \sum_{t=0}^{\infty} \binom{(u+1)\beta + t + u}{t} \alpha^u z^{t+u}, \tag{26}$$

$$= \sum_{u=0}^{\infty} (\alpha z)^u \sum_{t=0}^{\infty} \binom{(u+1)\beta + t + u}{t} z^t, \tag{27}$$

$$= \sum_{u=0}^{\infty} (\alpha z)^u (1-z)^{-(1+\beta)(u+1)} \tag{28}$$

$$= \frac{1}{(1-z)^{1+\beta}} \sum_{u=0}^{\infty} \left(\frac{\alpha z}{(1-z)^{1+\beta}} \right)^u \tag{29}$$

$$= \frac{1}{(1-z)^{1+\beta} - \alpha z} \tag{30}$$

I observe that $G(z)$ is only infinite when $(1-z)^{1+\beta} = \alpha z$. For real $0 \leq z \leq 1$, $0 \leq \alpha z \leq \alpha$ increases monotonically with z and $1 \geq (1-z)^{1+\beta} \geq 0$ decreases monotonically with z; and since $0 < 1$ and $\alpha > 0$, there is one unique intersection point between these for $z = r$. Let me repeat the definition $G(z) = \sum_{t=0}^{\infty} n(t) z^t$ and suppose I can approximate $n(t) \simeq n_0 g^t$ for some well chosen value g. For the right g, it counts $G(z)' = \sum_{t=0}^{\infty} n_0 g^t z^t \approx G(z)$. For the approximation to count for any z, I require that $g = 1/r$: if g would be smaller than $1/r$, then $gr < 1$ and $G(r)'$ would be a finite number where $G(r)$ is infinite; if g would be larger than $1/r$, $G(z)'$ would be infinite for $z > r$ where $G(z)$ would be finite. $G(z)$ is therefore only well approximated by $G(z)'$ if $g = 1/r$, in other words, a real number satisfying

$$(1 - 1/g)^{1+\beta} = \alpha/g. \tag{31}$$

Noting that $\alpha = g \left(1 - \frac{1}{g}\right)^{1+\beta}$, I observe that increasing α, keeping β constant, necessarily implies increasing g when β is kept constant, and vice versa. I can also rewrite Equation 31 as

$$\beta = \frac{\log g - \log(\alpha)}{\log g - \log(g-1)} - 1. \tag{32}$$

As $g > \alpha$ and $g > 1$, the numerator and denominator in this expression are always real and positive. When β increases, the fraction needs to increase. Differentiating the denominator with respect to g gives $1/g - 1/(g-1)$, which is always negative for $g > 1$. This implies that the denominator always decreases with increasing $g > 1$. Keeping α constant, the numerator only increases with increasing g. To increase the fraction in Equation 32 while keeping α constant, I necessarily need to increase g.

The number of classifications per patent

Finally, I calculate the number of classifications per family W using that, upon introduction, each family has on average W_0 classifications. Assuming W_0 is constant,

the number of unique patents is given by $\sum_c n_c(c)/W_0$, allowing me to write

$$W = \frac{n(t)}{\sum_{c=0}^{t} n_c(c)/W_0} \tag{33}$$

$$= W_0 \frac{n(t)}{\sum_{c=0}^{t} \alpha n(c-1)} \tag{34}$$

$$\approx W_0 \frac{n_0 g^t}{\sum_{c=0}^{t} \alpha n_0 g^{c-1}} \tag{35}$$

$$\approx W_0 \frac{g^t (g-1)}{\alpha (g^t - 1)} \tag{36}$$

$$\approx W_0 \frac{(g-1)}{\alpha} \tag{37}$$

Results for negative reclassification rates

The earlier analysis is mainly focused on the case where the net reclassification rate is positive, i.e. where $\beta > 0$. Here I explore what happens if $\beta < 0$ instead. While this case may be unlikely, it is conceivable that it applies to a particular class for some period of time. Let me first point out that nowhere in the model formulation I explicitly assume or need $\beta > 0$; the model outcomes are therefore equally valid for this case. This does not mean that the outcomes are always useful: when $\beta << 0$ and α is small, the number of patents in cohorts as predicted by Equation 18 hardly becomes greater than 1, meaning that, depending on the initial conditions, there is at some point no technological class left, or that the technology is unable to develop in the first place. The result are somewhat more interesting in the case where fast growth (large α) is combined with negative β. This case is plotted in Fig. 1. In line with what one expects for negative reclassification, I observe that patents are taken away from cohorts as t gets larger. However, after some time, the number of patents in cohorts remain stable. This stable part, analogous to the case where $\beta > 0$, shows exponential growth over the filing years with a growth factor as predicted by Equation 31. What is more striking: where there is a 'decline-time' for $\beta > 0$, here I observe a surge in the number of patents for recent cohorts instead. It is important to stress that this is no real surge: it is just that these patents have not yet been removed by reclassification. In conclusion, for negative β, the decline-time effect is reversed into an apparent surge of patenting in recent cohorts.

References

1. Graham RL, Knuth DE, Patashnik O. Concrete Mathematics: A Foundation for Computer Science. Reading: Addison-Wesley; 1989.

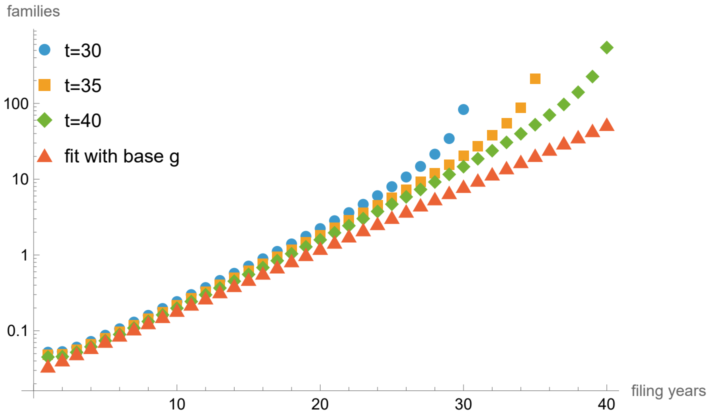

Fig 1. ***Model results for negative*** β. *Following the solution in Equation 18, this shows the number of patents in each cohort* $n_c(t)$ *plotted for each filing year* c *and for three values of time* $t = 30, 35, 40$. *In particular, I choose the values* $\alpha = 0.5$ *and* $\beta = -0.5$. *I also include an simple exponential relation* $\propto g^c$ *based on the same parameter values, using Equation 31. The number of patents increases exponentially until the apparent 'surge' when the filing year* c *of a cohort is close to* t, *that is, for recent cohorts.*
