# Supplementary material for: Can knowledge reclassification accelerate technological innovation?
Source: PLoS One. 2026 May 18;21(5):e0348804. doi: 10.1371/journal.pone.0348804 (PMC13183286; doi:10.1371/journal.pone.0348804)
Supplement: S2 Appendix — (PDF) [file pone.0348804.s002.pdf]

Supporting Information Appendix

Peter Persoon [1,2*¤]

1 Oxford Martin School, University of Oxford, United Kingdom
2 Wageningen University and Research, Wageningen, The Netherlands

¤Current Address: Wageningen University and Research, Droevendaalsesteeg 4, 6708 PB Wageningen
* peter.persoon@wur.nl

S2 Appendix: Empirical patterns

In this second Supporting Information Appendix I present in Section 1 the estimation of the model parameters α and β based on data. In Section 2, I include log log plot for the number of reclassifications over time. In Section 3, I include more background statistics of the positive, negative and net reclassifications. In Section 4, I include various plots to demonstrate the decline-time effect across different CPC sections and subclasses.

1 Estimating values for parameters beta and alpha

In this section, I will explain in more detail how the values for parameters β and α are estimated. As explained in the main text, I am applying the model to 'all technology', meaning that $n_c(t)$ represents all classifications of patents with filing year c at time t. For completeness, I repeat the data specifications: I consider patent families filed earliest between 1970 and 2019, with at least one US application and one in another jurisdiction. Using data from 4 Patstat editions (2013, 2016, 2019, and 2023), there are three distinct reclassification moments: 2013/2016,2016/2019 and 2019/2023. Reclassification happens yearly, so I aggregate the changes over approximately three years for each reclassification moment.

Recall the dynamics some technological class:

$$\Delta_t n_c(t) = \begin{cases} 0 & \text{if } c > t+1, \\ \alpha n(t) & \text{if } c = t+1, \\ \beta n_c(t)/(t-c+1) & \text{if } c < t+1, \end{cases} \tag{1}$$

where I denote the number of patents with filing year c at time t by $n_c(t)$ (i.e. the 'cohorts') and $n(t) = \sum_c n_c(t)$ (let $t = 0, 1, 2, 3, ..$ and $c = 0, 1, 2, 3, ..$). Here α and β are some positive constants that I would like to estimate.

Estimation of β (see Equation 1) is straightforward given the fits in Fig. 2 in the main text: given these three reclassification moments, I estimate an average value $\beta \approx 0.4$. These plots are based on reclassifications on the subclass level only. However, the value β are similar when considering different levels of classification. This is a result of using the net-reclassification per classification to determine beta, if the number of reclassifications per unique family were used, β would depend on the level of classification used.

Next, Equation 1 indicates that α could be measured by $\Delta_t n_c(t)|_{c=t+1}/n(t) = n_{t+1}(t+1)/n(t)$, i.e. by dividing the number of classifications of

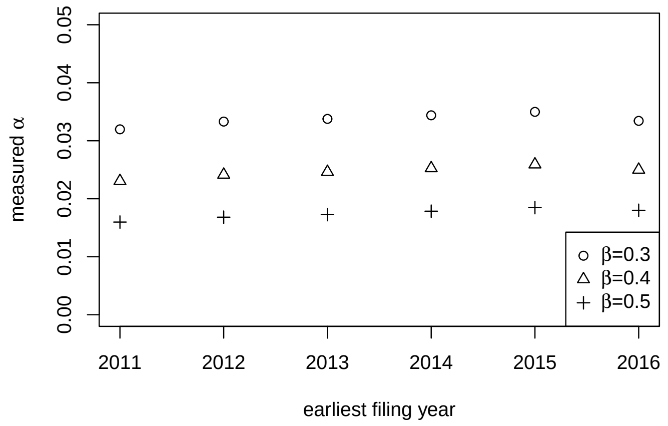

Fig 1. Estimated values for α between 2011 and 2016 based the number of classifications of patents in the 2023 data and then subtracting the expected number of reclassifications over the years for various estimated values of β.

newly introduced patents over the total number of classifications. Measuring this is challenging, however, because the number of newly introduced patents and classifications in recent years tends to be highly uncertain in patent databases, due to an (up to) 18-month period between the first filing and publication of a patent. For families with a much earlier filing year Y, however, the total number of classifications can reliably be measured; systematically reducing this number for each year between present and Y (using an estimate for β) thus allows me to estimate how many classifications there were at year Y (i.e., upon introduction). In other words, let me denote the number of classifications with filing year Y at year Y' by $C_{Y,Y'}$, then, given the present year P, the number of classifications upon introduction can be estimated by

$$C_{Y,Y} = C_{Y,P}\left(1-\frac{\beta}{1}\right)\left(1-\frac{\beta}{2}\right)...\left(1-\frac{\beta}{P-Y}\right) \quad (2)$$

Following 1, α can be calculated dividing $C_{Y,Y}$ by the total number of classifications of patents with filing years $< Y$ at year Y, i.e. $\sum_{Y'}^{Y} C_{Y',Y}$. The $C_{Y',Y}$ can be estimated again using Equation 2, more specifically,

$$C_{Y',Y} = C_{Y,P}\left(1-\frac{\beta}{Y-Y'+1}\right)\left(1-\frac{\beta}{Y-Y'+2}\right)...\left(1-\frac{\beta}{Y'+P}\right) \quad (3)$$

As this relation shows, the effect of transforming very early years (i.e. $Y' << Y$) is limited as the fraction $\frac{\beta}{Y-Y'+1}$ is then very small. Therefore, when estimating $C_{Y,Y}$ in the following, I only transform the years Y' for $Y' \geq Y-10$. The results of determining $C_{Y,Y}$ for $2011, 2012, 2013, 2014, 2015$, and 2016 for three estimated values of β are included in Fig. 1. Taking 2023 as present day, the values for $C_{Y,2023}$ are based on the 2023 dataset. The results indicate that the estimated values for α are rather stable of the years, justifying approximating it to be constant. Unsurprisingly, for greater β, the effect of reducing classifications is greater, therefore leading to smaller $C_{Y,Y}$, therefore leading to smaller estimates of α. Using the estimated value for $\beta \approx 0.4$ and classifications on the CPC group level, I estimate α to be between 0.024 and 0.027 (Fig. 1), on the CPC subclass level, these values are slightly lower, between 0.021 and 0.023.

Finally, the estimates for $C_{Y,Y}$ allow me to directly estimate W_0 i.e. is the number of the number of patents classifications per family upon introduction, by dividing the $C_{Y,Y}$ by the unique number of families in Y. For the years plotted in Fig. 1, I find that $1 < W_0 < 1.5$ on the CPC group level and $0.6 < W_0 < 0.8$ on the CPC subclass level. While values for $W_0 < 1$ may seem counter-intuitive, it should be stressed these estimates are based on a chosen, fixed value of β, which may not be a realistic assumption for the years considered.

2 Log-log plots empirical analysis

In line with Figure 2 in the main text, I again plot the number of reclassifications per year in Figure 2, except that I separately plot the negative and positive reclassifications, the horizontal axis is reversed, and both axes are log-transformed. Because the horizontal axes are reversed, the sharp increase towards the asymptote in Figure 2 in the main text is instead a sharp decline in Fig. 2, observable for both the negative and positive reclassifications. The number of classifications added tend to be larger than those deleted, except for the reclassification moment between 2013 and 2016 (the reasons for this remain unclear).

Furthermore, in Fig. 2 I include fits of the number of classifications added based on a simple inverse proportional relation with the number of years since reclassification. This relation, as any power law, appears as a straight line in this log-log plot. As the slope of the line is determined by the exponent (here -1). The data appear to follow the same slope, which suggests that the number of reclassifications are approximately inversely proportional with the number of years since reclassification.

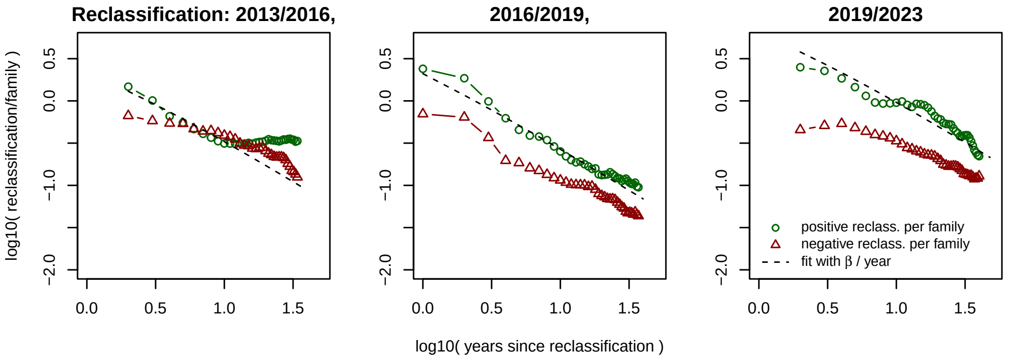

Fig 2. The number of positive and negative reclassifications per family per filing year on a log log plot, note the horizontal axis ('years since reclassification') is reversed in comparison to Fig. 2 in the main text. The slopes of the fits (included here for the positive reclassifications only) is -1, corresponding to an inverse relation with the time since reclassification.

3 Positive, negative and net reclassifications

Here I include a number of results relating more specifically to the distinction between positive, negative and net reclassifications on the CPC subclass level. For a given subclass x, the positive reclassifications rp_x are the number of patents obtaining a classification of a subclass x (and did not have one already), the negative reclassification are the number of patents loosing the classification of that subclass x, and the net reclassifications are simply $r_x = rp_x - rn_x$.

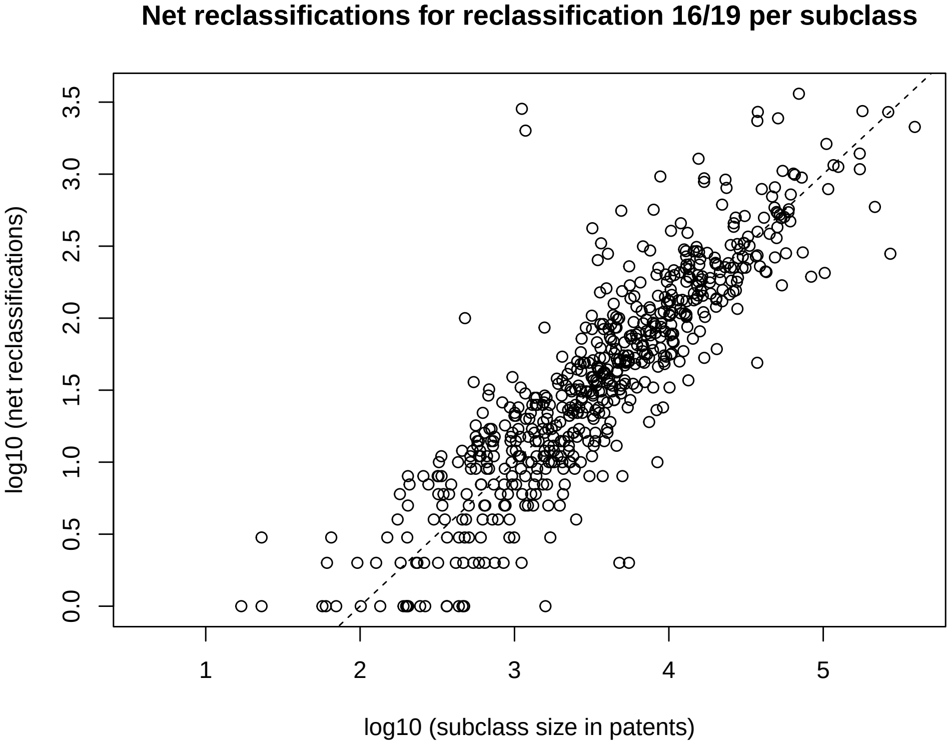

Fig 3. Here I plot the net reclassifications per subclass for the subclass size for reclassification moment 2016/2019. Note that both variables are log10 transformed to present a clearer picture, but as a consequence 21 subclasses (representing 3%) with negative net reclassifications are not included here. Included is also a dashed line with slope 1 (intercept -2), which forms an excellent fit with the data, suggesting a proportional relation between class size and net reclassifications.

For each reclassification moment considered in this research I include a number of descriptive statistics in Table 1. Here I observe that the positive, negative and net reclassifications generally have the same order of magnitude, although there are generally many more positive reclassifications. While these numbers appear substantial, they should also be perceived relative to the total number of subclass classifications. For example the positive reclassifications for the 16/19 moment represent about 1.6 percent of the total number of subclass classifications in 2016.

Table 1. Reclassification Summary

Reclassification moment moment	Positive reclassifications	Negative reclassifications	Net reclassifications
13/16	188932	54928	134004
16/19	125529	80279	45250
19/23	718839	166874	551965

To complement Figure 3 in the main text, I here include a Figure 3 where I plot the net number of reclassifications for subclass size for reclassification moment 16/19. Similar to the negative and positive reclassifications, the net reclassifications are observed to be proportional to subclass size. As the variables are log transformed in Fig. 3, the plot does not include those cases where the net reclassifications are negative. As it turns out, this is the case for 21 subclasses, representing about 3 percent of all subclasses. Surprisingly, when I sum the number of negative reclassifications of these 21 classes, I find it amounts to 62560 reclassifications: this is almost 78 percent of all negative reclassifications as can be verified in Table 1. This means that the negative reclassifications are highly concentrated in a small number of subclasses. In particular, I observe 25157, 16611, and 11546 negative reclassifications in respective subclasses Y02E, Y02B, and Y02P, indicating some heavy restructuring of the Y02 class.

Table 2. Reclassification descriptive statistics

Reclassification moment	mean rp	mean rn	mean rp without neg. subclasses	mean rn without neg. subclasses
13/16	291	85	289	38
16/19	192	122	182	28
19/23	1084	250	1064	50

These findings are further supported when I sub-select the subclasses with positive net reclassifications, and calculate the mean number of positive reclassifications and mean number negative reclassifications per subclass. These are included in Table 2 together with the mean values for the general case with all subclasses. Generally, the mean number of positive reclassifications are much larger than the negative ones, a pattern that becomes even more pronounced when removing the subclasses with net negative reclassifications. Especially for the 16/19 moment, the effect of removing the subclasses with net negative reclassifications results in a large correction into mean values of 182 positive versus 28 negative reclassifications per subclass. This reconfirms that, for that reclassification moment, even though the number of negative reclassifications are substantial, they are highly concentrated in a small number of subclasses.

Finally, I investigate if and how the total number of patents added to a subclass (dn) in a reclassification moment depends on the positive (rp) and negative (rn) reclassifications and controlling for subclass size (n). I include the OLS regression results for each reclassification moment 13/16, 16/19 and 19/23 in Tables 3, 4, 5. For each reclassification moment, a model is fitted including and excluding the negative

reclassifications. From all three analyses, it is clear that the relation between total patents added (indirectly relating to the growth factor of the subclass) and the negative reclassification is rather weak. While rn is statistically significant for the 16/19 and 19/23 moment, the model comparisons point out that including this variable hardly improves the R^2 of the fit and for the 16/19 moment, hardly improves the residual standard error. Of course, this is a rudimentary first analysis, but the outcomes suggest that, to better understand technology growth dynamics on a general level, there is no direct need to consider the negative reclassification as complementary to the positive - or net reclassifications. Of course, where the growth dynamics of individual technologies is considered for particular periods of time (see the earlier example with the Y02 class), considering the negative reclassifications in more detail may be very insightful.

Table 3. Estimation outcomes for models in- and excluding rn for the 13/16 moment

	Dependent variable:	
	dn	
	Full model	Restricted model
	(1)	(2)
rn	−0.203	
	(0.142)	
rp	1.878***	1.829***
	(0.091)	(0.085)
n	0.290***	0.290***
	(0.005)	(0.005)
Constant	−377.644***	−376.907***
	(92.986)	(93.059)
Observations	650	650
R^2	0.903	0.903
Adjusted R^2	0.903	0.903
Residual Std. Error	2,144.905 (df = 646)	2,146.631 (df = 647)
F Statistic	2,012.132*** (df = 3; 646)	3,012.327*** (df = 2; 647)
Note:		*p<0.1; **p<0.05; ***p<0.01

Altogether, this leads to the conclusion that, although the negative reclassifications are substantial in some cases, for an elementary analysis of overall technological growth dynamics, it suffices to work with the net reclassifications. Apart from special cases over specific periods of time, the number of net reclassifications per class or subclass is generally positive.

4 Time development of CPC sections and various subclasses

In this section, I include a number of plots with the number of patents (families) in different cohorts for four different versions of Patstat, first for CPC sections in Fig. 4, then for a selection of CPC subclasses in Fig. 5. I am interested in determining the

Table 4. Estimation outcomes for models in- and excluding *rn* for the 16/19 moment

	Dependent variable:	
	dn	
	Full model	Restricted model
	(1)	(2)
rn	-0.400^{***}	
	(0.095)	
rp	3.658^{***}	3.096^{***}
	(0.271)	(0.239)
n	0.093^{***}	0.096^{***}
	(0.005)	(0.005)
Constant	271.308^{**}	292.375^{**}
	(113.443)	(114.800)
Observations	654	654
R^2	0.709	0.701
Adjusted R^2	0.708	0.700
Residual Std. Error	2,669.789 (df = 650)	2,704.318 (df = 651)
F Statistic	528.163^{***} (df = 3; 650)	763.397^{***} (df = 2; 651)
Note:		*p<0.1; **p<0.05; ***p<0.01

decline-time, defined as the number of years between the Patstat-publication year (indicated by dashed lines, 2013, 2016 and 2019) and the year where the number of patents peak (which shifts to the right for different versions of Patstat). For example, for A61B, Patstat version 2013, the time development corresponding to Patstat2013 drops significantly after 2009 (four years before 2013), whereas, in the 2016 version, I observe an increase in the number of patents in 2009. But then the Patstat2016 development starts dropping after 2012 (four years before 2016), which is likewise shown not to be a real decline in the 2019 version, etc. For almost all considered cases, I analogously observe decline-times between 3 and 6 years long. This is larger than the reasonable delay of 2 years one can expect for any patent application before it is published. Note also that for some sections/subclasses and reclassification moments (for example section A, and subclasses E04B and H03M for the Patstat23 version), patents are added to very old cohorts, which is a typical sign of the subclasses being reclassified.

Fig 4. Here I plot the time development of CPC Sections according to different versions of Patstat (2013,2016,2019 and 2023). With dashed vertical lines the Patstat publication years 2013, 2016 and 2019 are indicated.

Fig 5. Here I plot the time development of various CPC subclasses according to different versions of Patstat (2013,2016,2019 and 2023). With dashed vertical lines the Patstat publication years 2013, 2016 and 2019 are indicated.

Table 5. Estimation outcomes for models in- and excluding rn for the 19/23 moment

	Dependent variable:	
	dn	
	Full model	Restricted model
	(1)	(2)
rn	-0.435^{***}	
	(0.042)	
rp	1.172^{***}	1.175^{***}
	(0.013)	(0.014)
n	0.127^{***}	0.106^{***}
	(0.004)	(0.003)
Constant	183.488^{*}	357.337^{***}
	(104.167)	(110.787)
Observations	663	663
R^2	0.948	0.940
Adjusted R^2	0.948	0.940
Residual Std. Error	2,439.982 (df = 659)	2,629.354 (df = 660)
F Statistic	$4{,}019.485^{***}$ (df = 3; 659)	$5{,}145.772^{***}$ (df = 2; 660)
Note:		*p<0.1; **p<0.05; ***p<0.01
