# Supplementary material for: Can knowledge reclassification accelerate technological innovation?
Source: PLoS One. 2026 May 18;21(5):e0348804. doi: 10.1371/journal.pone.0348804 (PMC13183286; doi:10.1371/journal.pone.0348804)
Supplement: S3 Appendix — (PDF) [file pone.0348804.s003.pdf]

Supporting Information Appendix

Peter Persoon [1,2*¤]

1 Oxford Martin School, University of Oxford, United Kingdom
2 Wageningen University and Research, Wageningen, The Netherlands

¤Current Address: Wageningen University and Research, Droevendaalsesteeg 4, 6708 PB Wageningen
* peter.persoon@wur.nl

S3 Appendix: Robustness checks and OLS results

In this third Supporting Information appendix, I discuss in detail three robustness checks as part of the model validation. Let me start with Fig. 1, where I plot the growth rates $g_k - 1$ for the number of classifications per patent w_k (on the CPC group level) for each group k similar to the plot in Fig. 6 in the main text, except I leave out the groups in subclasses C10M, C10N, B32B, and F17C, which have exceptionally large $w_k > 9$. Compared to the results in the main text, the correlations as measured by R^2

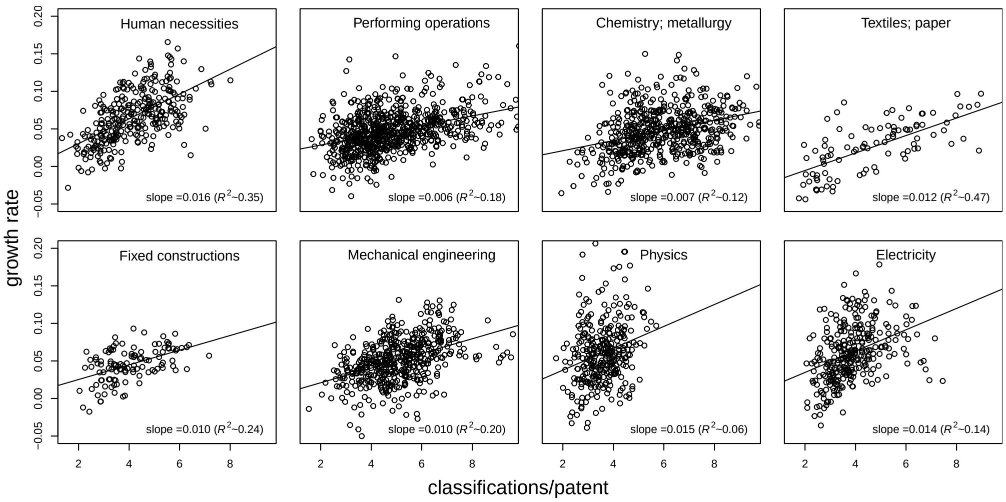

Fig 1. Growth rate $g_k - 1$ and number of classifications per patent w_k for each CPC group k, except the groups in subclasses C10M, C10N, B32B, and F17C.

in for the sections Performing Operations, Chemistry/metallurgy and Mechanical engineering are much higher, also visually the fits are much better. As the omitted subclasses have relatively large w_k, this could suggest that especially for lower w_k, the relation between $g_k - 1$ and w_k applies and might taper off for higher values. However, little can be said about this relation as the number of groups in C10M, C10N, B32B, and F17C are only a very small fraction of the total. In the following robustness checks, I focus on the relations without these subclasses, however, the results are still highly significant when including them.

Finally, I presents the details of three robustness checks I did on the observed positive relation between average classifications per patent W_k and the yearly growth

rate $g_k - 1$ of a CPC group k. As the strength of the relation may be rather different for different CPC sections, I analyze them separately [1]. I anticipate three criticisms:

1. That the observed relation between W_k and $g_k - 1$ may only be an indirect effect because both quantities would correlate with the size of CPC group k (in number of patents).

2. That the observed relation between W_k and $g_k - 1$ may only be an indirect effect because both quantities correlate to the characteristic to have many recent patents (this would count for W_k because reclassification especially targets recent patents, and many recent patents would bias $g_k - 1$ to greater values).

3. That the observed relation between W_k and $g_k - 1$ is only an 'apparent effect' because more reclassifications results in more classifications but not necessarily in more patents (or faster growth). This criticism suggests that if the $g_k - 1$ would be based not on the number of classifications but on the number of unique patents, there would not be a relation with W_k.

In the first robustness check, I address the first two criticisms by accounting for the (log) total group size n_k and the (log) number of patents in three recent years for each group in estimating a linear model using an Ordinary Least Squares (OLS). For the recent years I choose 2014, 2013 and 2012, as for any later year I am dealing with the apparent decline as discussed in the main text. The estimated relation is therefore summarized as

$$\begin{aligned} growth\_rate \propto & constant + class\_per\_family + log\_group\_total \\ & + log\_patents\_2014 + log\_patents\_2013 + log\_patents\_2012 \end{aligned} \tag{1}$$

The results are presented in Tables 1 and 2. From these tables it is clear that the relation between W_k and $g_k - 1$ is still robust after including the total number of patents in each group and the number of patents in recent years. Furthermore, I conclude that for most CPC sections, either the (log) number of patents in those recent years are not significant, or result in the fact the log group total number of patents are not (or no longer) significant.

In the second robustness check, I account for the second criticism in a different way. Instead of including the number of patents in the recent years as variables, I can also redefine W_k to make it 'less biased' to the number of patents in recent years: as there are relatively many reclassification in recent patents, if a group has relatively many recent patents, its average number of classifications per patent might be positively biased. To sidestep this, I slightly redefine W_k as the overall average of the yearly average of classification per patent (for each group k), which I will refer to as the 'year averaged classification per family' or 'year_av_class_per_family'. The second criticism would be justified if I would no longer find a significant relation between W_k and $g_k - 1$ after redefining W_k. The tested relation then becomes

$$growth\_rate \propto constant + year\_av\_class\_per\_family + log\_group\_total \ , \tag{2}$$

where for completeness I again account for group size. In Tables 3 and 4 the outcomes of the estimations are presented. I observe that there remains a highly significant positive relation between the growth rates and year_av_class_per_family, so even when I account for the bias brought to W_k by the number of recent patents, there is still a significant relation with the growth rates.

[1] I do not include the results for CPC section Y, which is a CPC wide tagging scheme to signal the potential of a technology to reduce greenhouse gas emissions and adapt to climate change. The Y classifications are however counted in the number of classifications per patent.

Table 1. OLS results classification per family and number of patents in recent years (A)

Section A

	Dependent variable:
	growth_rate
class_per_family	0.010***
	(0.001)
log_group_total	−0.014***
	(0.003)
log_patents_2014	0.006
	(0.004)
log_patents_2013	0.015***
	(0.005)
log_patents_2012	0.008*
	(0.004)
Constant	0.006
	(0.014)
Observations	300
R^2	0.728
Adjusted R^2	0.723
Residual Std. Error	0.018 (df = 294)
F Statistic	157.421*** (df = 5; 294)

Section B

	Dependent variable:
	growth_rate
class_per_family	0.003***
	(0.0004)
log_group_total	−0.019***
	(0.002)
log_patents_2014	0.005**
	(0.002)
log_patents_2013	0.009***
	(0.003)
log_patents_2012	0.014***
	(0.002)
Constant	0.052***
	(0.009)
Observations	752
R^2	0.532
Adjusted R^2	0.529
Residual Std. Error	0.019 (df = 746)
F Statistic	169.446*** (df = 5; 746)

Section C

	Dependent variable:
	growth_rate
class_per_family	0.003***
	(0.001)
log_group_total	−0.020***
	(0.002)
log_patents_2014	0.010***
	(0.003)
log_patents_2013	0.010***
	(0.004)
log_patents_2012	0.011***
	(0.003)
Constant	0.043***
	(0.011)
Observations	543
R^2	0.556
Adjusted R^2	0.552
Residual Std. Error	0.020 (df = 537)
F Statistic	134.303*** (df = 5; 537)

Section D

	Dependent variable:
	growth_rate
class_per_family	0.003***
	(0.001)
log_group_total	−0.027***
	(0.005)
log_patents_2014	0.021***
	(0.005)
log_patents_2013	0.001
	(0.005)
log_patents_2012	0.013***
	(0.004)
Constant	0.078***
	(0.026)
Observations	94
R^2	0.793
Adjusted R^2	0.782
Residual Std. Error	0.016 (df = 88)
F Statistic	67.615*** (df = 5; 88)

Note: *p<0.1; **p<0.05; ***p<0.01

Table 2. OLS results classification per family and patent numbers in recent years (B)

Section E

	Dependent variable:
	growth_rate
class_per_family	0.008***
	(0.001)
log_group_total	−0.023***
	(0.006)
log_patents_2014	0.021***
	(0.007)
log_patents_2013	0.009
	(0.006)
log_patents_2012	−0.002
	(0.006)
Constant	0.063**
	(0.024)
Observations	114
R^2	0.560
Adjusted R^2	0.540
Residual Std. Error	0.015 (df = 108)
F Statistic	27.511*** (df = 5; 108)

Section F

	Dependent variable:
	growth_rate
class_per_family	0.006***
	(0.001)
log_group_total	−0.023***
	(0.002)
log_patents_2014	0.012***
	(0.003)
log_patents_2013	0.008**
	(0.004)
log_patents_2012	0.011***
	(0.004)
Constant	0.047***
	(0.011)
Observations	478
R^2	0.618
Adjusted R^2	0.614
Residual Std. Error	0.018 (df = 472)
F Statistic	152.855*** (df = 5; 472)

Section G

	Dependent variable:
	growth_rate
class_per_family	0.009***
	(0.002)
log_group_total	−0.001
	(0.003)
log_patents_2014	0.003
	(0.006)
log_patents_2013	0.014**
	(0.007)
log_patents_2012	0.005
	(0.006)
Constant	−0.065***
	(0.016)
Observations	317
R^2	0.584
Adjusted R^2	0.577
Residual Std. Error	0.027 (df = 311)
F Statistic	87.214*** (df = 5; 311)

Section H

	Dependent variable:
	growth_rate
class_per_family	0.006***
	(0.001)
log_group_total	−0.010***
	(0.003)
log_patents_2014	0.009*
	(0.005)
log_patents_2013	0.004
	(0.005)
log_patents_2012	0.012**
	(0.005)
Constant	−0.003
	(0.014)
Observations	307
R^2	0.645
Adjusted R^2	0.639
Residual Std. Error	0.022 (df = 301)
F Statistic	109.333*** (df = 5; 301)

Note: *p<0.1; **p<0.05; ***p<0.01

Table 3. OLS results with year-averaged classification per family (A)

Section A

	Dependent variable:
	growth_rate
year_av_class_per_family	0.011***
	(0.001)
log_group_total	0.017***
	(0.001)
Constant	−0.100***
	(0.008)
Observations	300
R^2	0.598
Adjusted R^2	0.595
Residual Std. Error	0.022 (df = 297)
F Statistic	220.925*** (df = 2; 297)

Section B

	Dependent variable:
	growth_rate
year_av_class_per_family	0.004***
	(0.001)
log_group_total	0.014***
	(0.001)
Constant	−0.069***
	(0.006)
Observations	752
R^2	0.336
Adjusted R^2	0.334
Residual Std. Error	0.022 (df = 749)
F Statistic	189.633*** (df = 2; 749)

Section C

	Dependent variable:
	growth_rate
year_av_class_per_family	0.004***
	(0.001)
log_group_total	0.015***
	(0.001)
Constant	−0.085***
	(0.009)
Observations	543
R^2	0.289
Adjusted R^2	0.287
Residual Std. Error	0.026 (df = 540)
F Statistic	109.954*** (df = 2; 540)

Section D

	Dependent variable:
	growth_rate
year_av_class_per_family	0.011***
	(0.001)
log_group_total	0.015***
	(0.003)
Constant	−0.120***
	(0.023)
Observations	94
R^2	0.490
Adjusted R^2	0.479
Residual Std. Error	0.025 (df = 91)
F Statistic	43.778*** (df = 2; 91)

Note: *p<0.1; **p<0.05; ***p<0.01

Table 4. OLS results with year-averaged classification per family (B)

Section E

	Dependent variable:
	growth_rate
year_av_class_per_family	0.008***
	(0.002)
log_group_total	0.011***
	(0.002)
Constant	−0.060***
	(0.015)
Observations	114
R^2	0.360
Adjusted R^2	0.349
Residual Std. Error	0.018 (df = 111)
F Statistic	31.264*** (df = 2; 111)

Section F

	Dependent variable:
	growth_rate
year_av_class_per_family	0.006***
	(0.001)
log_group_total	0.017***
	(0.001)
Constant	−0.093***
	(0.011)
Observations	478
R^2	0.282
Adjusted R^2	0.279
Residual Std. Error	0.025 (df = 475)
F Statistic	93.329*** (df = 2; 475)

Section G

	Dependent variable:
	growth_rate
year_av_class_per_family	0.009***
	(0.003)
log_group_total	0.022***
	(0.001)
Constant	−0.137***
	(0.015)
Observations	317
R^2	0.424
Adjusted R^2	0.420
Residual Std. Error	0.032 (df = 314)
F Statistic	115.556*** (df = 2; 314)

Section H

	Dependent variable:
	growth_rate
year_av_class_per_family	0.008***
	(0.002)
log_group_total	0.018***
	(0.001)
Constant	−0.110***
	(0.011)
Observations	307
R^2	0.458
Adjusted R^2	0.454
Residual Std. Error	0.027 (df = 304)
F Statistic	128.196*** (df = 2; 304)

Note: *p<0.1; **p<0.05; ***p<0.01

In the third and last robustness check, I address the earlier mentioned third criticism. According to that criticism, the boosting of growth rates of groups with many reclassified patents is not a real acceleration because the reclassified patents are only 'copies', i.e. they already exist elsewhere in another group. To counter this criticism, I slightly redefine the growth rate (and group totals) such that it only counts each unique patent once. If a patent is classified in multiple groups, I divide it evenly over these groups, i.e. I do a fractional attribution of patents across groups. I will refer to resulting growth rate and group totals as 'growth_rate_fractional' and 'group_total_fractional'. The third criticism would be justified if I would no longer find a significant relation between W_k and $g_k - 1$ after redefining the growth rate as such. The tested relation therefor becomes

$$growth\_rate \propto constant + \; year\_av\_class\_per\_family + log\_group\_total \; , \qquad (3)$$

where for completeness I again account for group size. In Tables 5 and 6 the outcomes of the estimations are presented. I observe that there remains a highly significant positive relation between the redefined growth rates and classification per family.

Table 5. OLS results with fractional patent counting (A)

Section A

	Dependent variable:
	growth_rate_fractional
class_per_family	0.015*** (0.001)
log_group_total_fractional	0.018*** (0.001)
Constant	−0.118*** (0.009)
Observations	300
R^2	0.573
Adjusted R^2	0.570
Residual Std. Error	0.023 (df = 297)
F Statistic	198.875*** (df = 2; 297)

Section B

	Dependent variable:
	growth_rate_fractional
class_per_family	0.007*** (0.0005)
log_group_total_fractional	0.012*** (0.001)
Constant	−0.071*** (0.007)
Observations	752
R^2	0.294
Adjusted R^2	0.292
Residual Std. Error	0.024 (df = 749)
F Statistic	156.150*** (df = 2; 749)

Section C

	Dependent variable:
	growth_rate_fractional
class_per_family	0.008*** (0.001)
log_group_total_fractional	0.013*** (0.001)
Constant	−0.091*** (0.010)
Observations	543
R^2	0.240
Adjusted R^2	0.237
Residual Std. Error	0.028 (df = 540)
F Statistic	85.203*** (df = 2; 540)

Section D

	Dependent variable:
	growth_rate_fractional
class_per_family	0.014*** (0.001)
log_group_total_fractional	0.014*** (0.004)
Constant	−0.130*** (0.025)
Observations	94
R^2	0.498
Adjusted R^2	0.487
Residual Std. Error	0.027 (df = 91)
F Statistic	45.202*** (df = 2; 91)

Table 6. OLS results with fractional patent counting (B)

Section E

	Dependent variable:
	growth_rate_fractional
class_per_family	0.010^{***}
	(0.002)
log_group_total_fractional	0.012^{***}
	(0.002)
Constant	-0.076^{***}
	(0.016)
Observations	114
R^2	0.339
Adjusted R^2	0.327
Residual Std. Error	0.019 (df = 111)
F Statistic	28.428^{***} (df = 2; 111)

Section F

	Dependent variable:
	growth_rate_fractional
class_per_family	0.011^{***}
	(0.001)
log_group_total_fractional	0.013^{***}
	(0.001)
Constant	-0.094^{***}
	(0.010)
Observations	478
R^2	0.302
Adjusted R^2	0.299
Residual Std. Error	0.025 (df = 475)
F Statistic	102.613^{***} (df = 2; 475)

Section G

	Dependent variable:
	growth_rate_fractional
class_per_family	0.019^{***}
	(0.003)
log_group_total_fractional	0.022^{***}
	(0.001)
Constant	-0.174^{***}
	(0.015)
Observations	317
R^2	0.444
Adjusted R^2	0.440
Residual Std. Error	0.033 (df = 314)
F Statistic	125.184^{***} (df = 2; 314)

Section H

	Dependent variable:
	growth_rate_fractional
class_per_family	0.018^{***}
	(0.002)
log_group_total_fractional	0.017^{***}
	(0.001)
Constant	-0.131^{***}
	(0.011)
Observations	307
R^2	0.470
Adjusted R^2	0.466
Residual Std. Error	0.028 (df = 304)
F Statistic	134.727^{***} (df = 2; 304)

Note: $^{*}p<0.1$; $^{**}p<0.05$; $^{***}p<0.01$
