# Supplementary material for: Can knowledge reclassification accelerate technological innovation?
Source: PLoS One. 2026 May 18;21(5):e0348804. doi: 10.1371/journal.pone.0348804 (PMC13183286; doi:10.1371/journal.pone.0348804)
Supplement: Attachment — Submitted filename: Reponse to reviewers.pdf [file pone.0348804.s005.pdf]

Dear editor, dear reviewers,

Thank you very much for the opportunity to revise my manuscript about technological reclassification and growth dynamics. I am very grateful for the comments and suggestions: they are very spot on, demonstrate some deep thinking, and even make very original suggestions for the model improvement. Below I have addressed each point you made. There was some overlap in the points you raised, in those cases I have tried to systematically refer to the points where the issue was dealt with. I include a version of the manuscript with track-changes, where the added text is highlighted in **bold.** Note that I often refer to line/row numbers in the manuscript file with annotations .

Best wishes,

Peter Persoon

Points raised by the editor

1. Editor: *Help readers who may be less deeply familiar with the patent process to understand the phenomenon you are studying. Perhaps provide some examples of patents that have been reclassified to make this phenomenon more concrete. Relatedly, explain why you focus on patent families, rather than individual patents, and what the difference is both conceptually and empirically.*

   Reponse: The editor raises two valid points about the meaning of reclassification and patent families. The old version went over these aspects too quickly. In the new version I provide a better explanation of process of reclassification and what patent families are, more precisely

- On Lines 80-93, I have added a better explanation of process of reclassification including a concrete example of a reclassified patent (patent US4671271). I point out what this means for the individual patent and the classes it is in. Finally, I added a small digression on possible reasons for reclassification and the empirical challenges connected to this.

- On Lines 102-108, I added short motivation for using patent families followed by an explanation of what patent families are. Apart from a general definition, I add the empirical definition used in this research which is based on docdb family id.

2. Editor: *Be clearer at the outset about what the contribution of your article is. This is really the central concern of reviewer #2. I think that addressing it would go some way to placating the concerns of reviewer #1 as well. In particular are you only trying to develop a mathematical model that fits the observed patterns? Or are you trying to explain something about the underlying processes that generate these patterns? If the latter, then there is an inherently causal part of your story, and you need to grapple with issues of causality. I think the reviewers read this differently and respond differently as a result.*

   Response: The editor and both reviewers rightly point out that the old version is somewhat unclear about the contribution of the paper. I also agree that the conclusion that knowledge development could be accelerated with more extensive reclassification

is somewhat overstating the results, especially as long as issues of causality have not been appropriately dealt with. While I had hoped a warning about causality in the introduction and discussion would suffice, I now appreciate that the paper should be clearer in this respect. Determining causality is not the ambition in this work, the aim is to show how integrating simple reclassification and triggering processes (that are grounded by empirical patterns) in a single mathematical model lead to a non-trivial relation between reclassification and growth rates. Even without determining the exact causal processes underlying these relations, the model leads to predictions about other knowledge quantities which can again be tested empirically.

Again I agree that this could have been made much clearer in the old version. I therefore decided to substantially rewrite and make additional statements throughout various parts of the paper (here organized by section):

Abstract:

- I deleted the suggestive sentence about renewed sources of innovation and instead wrote a more neutral sentence of how the paper investigates the relation between reclassification and growth.
- I deleted a sentence that suggests that the model explains ‘how reclassified invention contribute to faster innovation’. Instead I added a sentence explaining how I integrate empirical patterns into an analytical model that allows for a detailed description of the relation between reclassification and growth rates.

Introduction:

- Lines 12-15: Similar to the change in the abstract, I removed suggestive language about renewed sources and instead write a more neutral sentence about the reclassification effect and it general dynamics.
- Lines 22-27: Deleting a short and unclear description about the contribution of the paper and instead adding a more detailed explanation of the aim of the paper.
- Lines 32-25: Reformulation to stress that causality is still uncertain and remove suggestive language of ‘lever to accelerate’.
- Line 39: Reformulation of terms that are more neutral from the perspective of causality.

Analytical part:

- Line 176: rewrote sentence to make the intention of the model more clear (which is to simply describe the interaction mathematically).
- Lines 228-235: removed suggestive language about causality (e.g. ‘leads to’) an instead chose a more neutral formulation of the mathematical relation.

Discussion:

- Lines 369-372: Rewrote sentence such that the focus is not on the positive relation between reclassification and growth rate but on the empirical support of the model.
- Line 376: more emphasis on limitations
- Lines 387-389: with special thanks to the suggestions of reviewer #2, I added a sentence here about the possibility of indirect causation and unknown variables affecting both reclassification and growth.

Finally, I have also considered changing the title of the manuscript. However, since it is in a question form, it leaves open the possibility that another scenario might apply. I hope you allow me to keep it as it is.

3. Editor: *I am inclined to agree with reviewer #1 that the model terminology is difficult to follow and encourage to consider whether it can be made easier to follow, and that more intuition provided.*

   Response: I agree that the write-up of the analytical model is somewhat compact in the old version, of course it was certainly not the intention to make it more difficult then necessary. In the new version, I have made a number of changes to the model formulation, where I focused on a more intuitive explanation of the dynamics, take away confusion in the time-related variables, and create a clearer text structure for calculated quantities. I would like to thank reviewer #1 for the suggestion to use the concept of cohorts, which I have given a central role in this new version. More specifically, I made the following changes:

- On Line 149: a first mentioning of cohorts in the empirical part to keep consistency with analytical part.
- Line 181: a better explanation of cohorts, mention time dependence of cohorts more explicitly
- Line 189: added phrase to make more explicit when the first part of the dynamics is applicable
- Line 198: added phrase to make more explicit when the second part of the dynamics is applicable
- Line 201: added phrase to make more explicit when the third part of the dynamics is applicable.
- Line 203 (or Equation 1): here I reversed the organization of cases, where in the old version it was organized for different cases of time t, in the new version it is organized by cohort cases. This organization is more intuitive for a given technological class.
- Line 229 and 234: a less mathematical, more intuitive explanation of model consequences. I rewrote the phrases suggesting a causal relation here.
- Line 246: here, instead of using T to denote the decline time, I introduce the notation t_{d} to make a clearer reference to the decline aspect.
- Line 237: introduction of structure for the three calculated quantities, furthermore, subsection headers are included for these quantities.
- Caption Fig 4: reformulation in terms of cohorts and a number of further clarifications.

4. Editor: *Again, like reviewer #1 I am puzzled that you are only modeling reclassifications into a class. But presumably all reclassifications must also be the result of a reclassification out of some other class. Shouldn't you be modeling both of these phenomena?*

   Response: This response consists of three parts,

   - first a clarification about net reclassification,

- second an explanation of an additional analysis of negative reclassification
- third an explanation of an additional analysis (empirically) of including negative positive reclassifications in parallel for different subclasses.

Clarification about net reclassification

The editor and reviewer #1 raise a valid point that, in the old version, it is not sufficiently clear how the reclassification out of some class, also referred to as 'negative reclassifications' is incorporated in the analytical model. The explanation should have been that the rate of reclassification β should be interpreted as a 'net reclassification rate' (as reviewer #2 rightly did). Since the positive and negative reclassifications (according to both the empirical analysis in section 2 and the model assumptions) act completely analogously on the cohorts, subtracting them in the rate β is justified. Again I confess that this aspect was not made clear in the old version, which is why made the following changes to the main text:

- Line 150: more explicitly introduce β as 'net reclassification rate'
- Line 152: explicitly write down the possibility of a positive and negative net reclassification rate β.
- Line 195: mention also the possibility of reclassification out of a class.
- Line 196: add sentence clarifying the model works with a net reclassification rate.

Additional analysis of negative reclassification

The editor and reviewers' remark about the negative reclassification is valuable also because it begs the question: what if the net reclassification rate becomes negative? Though unlikely, this might be a possibility for a given class and/or for a given period of time. In the old version this possibility was completely ignored, which is another reason for confusion perhaps. I therefore added a remark about this in the main text on

- Line 205: Here I mention generality for any β but also that I focuse on the positive β case. I refer to appendix for a short discussion of negative β case.

Thus, I created an extra section in the SI appendix (Section 1.4) where I discuss the negative β case. To create this I first reviewed the model to explore the implications for negative values of β and plotted some results. I found that the model formulation nowhere relies on the assumption that β has to be positive, so the model predictions should be accurate for this case as well. That does not mean that the prediction are always useful: in the case of low α (slow growth) and negative β with absolute value much greater than α, the number of patents does not go above 1 (which basically means we have no technology class left anymore). For parameter ranges where α is large (fast growth) and β is negative (with absolute value comparable to α), the number of patents develop in a non-trivial fashion. To show this I include a plot including a plot for an example of these parameter values. In short, in the latter parameter range, we still observe exponential growth (with growth factor g as predicted), but now instead of a 'decline time', we actually see that toward recent years, the number of patents sharply increase. In this case, the model thus predict complete reversal of the 'decline time' effect.

Additional empirical analysis with positive and negative reclassification in parallel
Next to investigating the consequences of negative net reclassification, another interesting angle would be investigate the added value of treating negative reclassification separately from positive reclassifications in their relation to growth dynamics. This angle I believe relates more closely to what Reviewer #1 intends in point his/her third point. Indeed this point motivated me to do an additional analysis treating the two types of reclassifications separately, I report about this in my response to point 3 raised by Reviewer #1 and in the SI appendix Section 4.

Points raised by Reviewer #1

1. Reviewer #1: *I tried very hard to understand the model and its empirical motivation, but the use of terminology made this unnecessarily difficult. In particular, the indexing of time dimensions with t and tau (and occasionally other terms) was extremely hard to follow. I repeatedly had to go back and check whether t was filing year, observation year, or both. This could be dramatically improved by adopting more standard temporal indexing: for example, filing year or cohort year (c), and observation or calendar year (t). More generally, I think the model would be much easier to interpret if each variable had an intuitive label and was described clearly in plain language.*

   Response: I hope to have addressed these criticisms in my response to point 3 raised by the editor. Note in particular how the concept of cohort now plays a central role (for which I am grateful), how I tried to modify the model explanation such that is more intuitive and clear and finally how the time-related quantities are relabeled.

2. Reviewer #1: *A central empirical finding of the paper is that patents are both added to and removed from classes over time. But the model only includes additions. There is no representation of reclassification out of the focal class. This is a major omission. Any generative model of class-level growth needs to account for outflows, not just inflows. As it stands, the model is not just empirically incomplete, but is logically flawed.*

   Response: I hope to have addressed these criticisms in point 4 raised by the editor.

3. Reviewer #1: *That said, the omission of reclassification-out is actually a huge opportunity. I would suggest the author reframe the paper as a decomposition exercise, and formally define three processes: alpha (endemic growth), beta (reclassification into), and omega (reclassification out of). The net change in class size over time would then be:*

   delta_n = alpha + beta – omega

   This is a straightforward accounting identity, and it opens the door to richer empirical comparisons across classes. For example, some classes may grow mainly through alpha (internal invention), others through beta (absorbing work from elsewhere), and others may shrink through omega (having their content absorbed by other domains). These distinctions could then be linked to theoretical expectations from the innovation literature—for example, about greenfield invention vs. recombination vs. obsolescence.

Response: I really appreciate this suggestion by Reviewer#1 which demonstrates some deep thinking about the subject. This point motivated me to dive back into the data and perform various additional analyses. I focused mainly on the question: what can we learn empirically from the negative reclassifications, considering them as complementary to positive reclassifications, in relation to growth dynamics?

I started by determining the positive and negative reclassifications on the subclass level for each reclassification moment (i.e. 13/16, 16/19 and 19/23). In line with Reviewer #1's suggestion, I started by calculating correlations and doing simple OLS regressions between total added number of patents and the number of negative reclassifications, positive reclassifications, also including subclass size and average filing year. To my surprise, where the positive reclassifications were always highly significant, I only found weak relations between the negative reclassifications and the total added patents. Between positive and negative reclassifications I found a moderately strong correlation. From my earlier analysis it was already known that both the negative and positive reclassifications relate positively to class size. For a more detailed discussion of these results I refer to SI Appendix Section 4.

Of course the fact that the negative reclassification do not seem to play a significant role (complementary to positive reclassifications) in relation to growth dynamics does not mean that they are not worth studying. As Reviewer #1 rightly points out, there may be interesting variations in the degree of negative reclassification across technological classes that can possibly be related to various concepts in the innovation literature. The focus of this work was however the relation between reclassification and growth dynamics, in which the negative reclassifications after this first scan appear to play a limited role. I therefore decided to keep these findings for the supplementary material (see SI Appendix Section 4) and to not pursue this path for now. However, the reader may want to know about the possibility of using negative reclassifications. I therefore include a reference to this analysis in the main text:

- On lines 130-135: Reference to analysis in SI about negative and net reclassifications.

4. Reviewer #1: *The current write-up strongly implies causal mechanisms that are not identified. I am not a stickler on causality, but I still found the causal language to be really problematic.*

   Response: I hope to have addressed this in point 2 raised by the editor, which involved as substantial revision of the causal language.

5. Reviewer #1: *The idea that the drop-off in recent-year patent counts is an artifact of delayed reclassification is plausible, but not appropriately tested. We would need to track cohorts of patents forward over time and show that their number of class assignments continues to grow, and that the growth is disproportionately concentrated in certain classes. Figure S3 in the supplement provides some evidence in this direction, but it's aggregate and not disaggregated by class.*

Response: Reviewer #1 raises a valid point that in the old version, the recent-year drop-off is only demonstrated empirically for the aggregate classifications and not separately for certain classes. I agree that the demonstration of this effect would be more convincing if these patterns are demonstrated in a systematic way for various classes (for each cohort and for each classification version). For this reason I added a number of plots in the SI Appendix Section 5 where the effect is also demonstrated on the CPC section level and for 6 subclasses. These subclasses were chosen more or less randomly. I refer to these plots in the main text:

- On lines 312-315: when discussing the validation of the decline-time, I refer to the extra plots in the SI Appendix.

As I explain in more detail in SI Appendix Section 5, the substantial decline times are also clearly observable for the various sections and subclasses separately. Furthermore, I observe patents added to very old cohorts (over 10 years old), which is a typical aspect of reclassification.

6. *Figure 5 is treated as empirical validation of the model in Figure 4, but it relies on the assumption that changes in patent-class assignments reflect reclassification rather than administrative changes or late additions. Without more careful documentation of the underlying data structure, this assumption is hard to verify.*

Response: Reviewer #1 raises a valid point that applies more generally to the way reclassification is here investigated: by comparing different editions of the patent classification system, we do not see the underlying reasons for changes, only the changes itself. While an important shortcoming of the current research-set up, I cannot see a straightforward way to solve this. If one would study the reclassification changes within one group or subclass, it makes sense to try and look up the administrative changes that had an impact on it, but for the aggregate (consisting of more than 600 subclasses and 10000 main groups), this quickly becomes intractable. In this research, instead of diving into the specifics of a certain class (where understanding the reasons for reclassification is vital), I tried to keep the bigger picture and describe the general dynamics (for which the various, intertwined reasons for reclassification are perhaps less important). This point was perhaps not made sufficiently clear in the old version of the manuscript, in the new version I include a statement about this:

- On line 90: added statement about the difficulty of understanding the reasons for reclassification

Finally, I hope to have countered some of the data-quality issues (possibly caused by administrative errors) by selecting families that have more than one member and a US-member. These are commonly used filters to exclude questionable patents.

7. Reviewer #1: *The supplement is helpful in clarifying that most patents gain more classes than they lose. But again, this is shown in the aggregate. The model is applied at the class level, so ideally the author would provide class-level measures of inflows and outflows, and possibly their balance.*

Response: The reviewer rightly points out that figures such as Fig. 3 in the manuscript present some information about negative and positive reclassification but give little clarity about how these would balance out for various subclasses. To fix this, I did an additional analysis about which I report in SI appendix Section 4. In this analysis I dive somewhat deeper in the question how the negative and positive reclassifications are divided over the CPC subclasses. In SI Section 4 I include a figure (Fig. 5) in which, analogous to Fig. 3 in the main text, the net reclassifications are plotted for 97 percent of the subclasses. This allows for a direct observation of the balance, which follows the patterns of the positive reclassifications rather closely. The remaining 3 percent have negative net-reclassification and are further studied in this section. Summarizing the main findings here, the negative reclassifications appear to be highly concentrated in a small number of subclasses, some of which may have negative net reclassifications. Excluding these subclasses from the analysis leads to the insight that for most subclasses, the number of positive reclassifications is much greater than the negatives ones.

A reader might want to know about this. I hope to have covered that in the modifications to the main text described in the response to point 3 raised by Reviewer #1.

8. Reviewer #1: *A very interesting interpretive possibility, not pursued in the current manuscript, is that classes with high omega values (reclassification out of the focal class) are being "liquidated" or conceptually repurposed—for example, they are no longer meaningful categories, and their content is being absorbed by newer or adjacent domains. That is an interesting phenomenon in its own right and could be explored more directly.*

   Response: Again Reviewer #1 raises a very interesting point. Some of these effects I may have encountered in the analysis described in the (new) Section 4 in the SI Appendix. In this analysis, I noticed that the negative reclassifications are rather concentrated in a small number of subclasses, where I also name some examples: subclasses Y02E, Y02B and Y02P. To be sensible about these large restructuring events however really requires us to get in touch with the patent office or dive deeper into their classification documentation, which would suggest a rather different angle to this research (relating to the discussion following point 6 raised by Reviewer #1). To go in this direction is however not the ambition of this contribution but is certainly promising for a follow-up.

   Again I refer to the response to point 3 to demonstrate how I modified the main script to better account for the negative reclassifications, to which this closely relates.

9. Reviewer #1: *The model assumes that alpha and beta are separate processes. But the paper does not really discuss whether they are driven by different underlying mechanisms. This relates to the thinness of any theory in the paper. Could certain types of technologies be more likely to attract reclassifications? Could others be more likely to retain their boundaries?*

   Response: I really appreciate the suggestion by Reviewer #1 to think about what type of technologies would be particularly sensitive to the act of reclassification and similarly if

there is some typification possible for technologies in which the triggering mechanism plays an important role. One type I can think of for the former dimension is the idea of general purpose technologies: their great spectrum of applicability might along various lines connect to many other inventions (some possibly older than the general purpose technology), which may then be reclassified as part of the general purpose technology and thereby further extending its spectrum. Underlying these dynamics is also a 'rich get richer' mechanism which possibly connects to the finding of the paper that larger classes attract more reclassifications.
While it would be very interesting to directly link reclassification and 'multi-purposeness', the attempt of measuring or quantifying the latter is a paper in itself. However, as suggested by Reviewer #1, it is valuable to report about these possible theoretical connections. I modified the text both in the Introduction (the most natural place to make these connections) and in the parts of the paper where these concepts are used. To be precise, I modify the main text:

- On line 39: explain the connection with technological scope and general purpose technologies.
- On line 153: make more explicit where the idea of a wider scope enters the model reiterate .

The second dimension, that of triggering, is closely related to how 'cumulative' a technology is, i.e. the extent to which it depends on earlier building blocks to further develop. I agree with the Reviewer that in the old version, the link with the cumulativeness literature is underrepresented. I therefore modified the text

- On line 49: explain the relevance of this paper to the cumulativeness literature
- On line 185: make the link between the model and patterns of cumulativeness more explicit.
- On line 379: again explicitly mention the cumulative mechanism of knowledge development to emphasize the link with this literature.

10. Reviewer #1: *The paper needs to better articulate its contribution to the innovation literature. I found the empirical pattern interesting and the proposed decomposition compelling, but I was not always sure what research question the author was trying to answer. Is the paper trying to explain technological change? The evolution of classification systems? The role of recombination? A more direct statement of the paper's aims would help a lot. As written, the paper sits between description and theory, and it's not clear what the key takeaways are meant to be. In particular, there is no theory discussed in the paper. If the author believes that reclassification is associated with growth, it would be helpful to understand the theoretical basis for that belief. Why should we expect reclassification to be positively associated with a class's growth over time?*

    Response: In this point, Reviewer #1 sums up a number of criticisms, some of which I hope to have covered in earlier points raised by both the editor and Reviewer 1. In the following I will try to make clear where and how these criticisms are addressed. First there is the criticism about better articulating the added value of this contribution to the

innovation literature and related, what research question I am trying to answer. I hope to have addressed this in my response to point 2 raised by the editor.

Then there is the criticism about the key takeaways. I agree that the abstract could include these more clearly. I therefore decided to rewrite the end of the abstract such that it now includes more clearly the core implications:
'In sum, the model allows for a better understanding of both the magnitude and shape of technological growth patterns. More generally, it connects various, seemingly unrelated knowledge quantities, providing a basis for knowledge intrinsic explanations of growth patterns.'

Finally there is the criticism about discussing theory in the paper, where I assume Reviewer#1 means proposing a mechanism and connecting that to ideas in the literature. The mechanism I hope to convey with this paper is the simple combination of triggering and (cohort specific) reclassification, yet I think the Reviewer has a valid point that these interactions could have been linked better to existing literature. I hope to have covered this in my response to point 9 raised by Reviewer #1, where I made a number of further connections with the literature which I also bring up in the Introduction. Finally, I strongly believe that reclassifications is a highly understudied subject, implying that such connections in the literature may still be much underdeveloped. Hopefully this contribution is a step in that direction.

Points raised by Reviewer #2

1. Reviewer 2: *I have one request, before moving on to some general comments. I think the model could be clearer about how one should think about positive and negative reclassifications. I have interpreted the model as describing the net effect of positive and negative reclassifications, but I would have appreciated a more explicit steer here, on page 5. I found it a bit puzzling to document positive and negative reclassifications, but then to ignore this dynamic when setting up the model. This is my only substantive request for a change, and I think it is a minor one.*

   Response: I hope to have addressed this point in my response to point 4 raised by the Editor.

2. Reviewer 2: *I think thorny challenges remain around interpreting the significance of this result. I do not believe the paper has shown reclassification to be causal, and my null hypothesis is that the underlying dynamics are instead driven by two unobserved factors. First, new technological types arise frequently and are often a poor fit to existing classifications. These new types vary in their importance. When a new type is more important, more inventors develop inventions and seek patents for inventions of that type. More frequent contact with patent applications of the new type creates more pressure to properly assign patents of this new type to an appropriate technology class. So you get a correlation between the number of reassignments and the number of new patents sought in a class. Meanwhile, these effects might be amplified by a learning process, wherein patent examiners decide how to initially assign or later reassign a patent to a class based on the class assigned to similar patents. A patent's most similar*

match is most likely to belong to a class with more rather than less patents, which creates an additional rich-get-richer effect.

Response: I largely agree with Reviewer #2 on this issue and I hope my response to point 2 raised by the editor shows how I addressed it in this review. I greatly appreciate the thoughts shared by Reviewer #2 on the issue.

Additional changes not related to editor/reviewer remarks:

- I altered the Acknowledgement section to express my appreciation for points the editor and reviewers raised.
- There was an error in the caption of Fig 2., where in the old version it said β=0.5, it should be 0.7.
