# Supplementary material for: Can knowledge reclassification accelerate technological innovation?
Source: PLoS One. 2026 May 18;21(5):e0348804. doi: 10.1371/journal.pone.0348804 (PMC13183286; doi:10.1371/journal.pone.0348804)
Supplement: Attachment — Submitted filename: rebuttal letter.pdf [file pone.0348804.s006.pdf]

Dear editor, dear reviewers,

Thank you very much for the positive message that all the points were appropriately dealt with. I hereby upload a version where the final errors in the make-up should be corrected.

I would also like to thank you again for your valuable feedback,

Best wishes,

Peter Persoon
